# Serum cyclin-dependent kinase 9 is a potential biomarker of atherosclerotic inflammation

**DOI:** 10.18632/oncotarget.6443

**Published:** 2015-12-01

**Authors:** Yeming Han, Shanshan Zhao, Yaoqin Gong, Guihua Hou, Xi Li, Li Li

**Affiliations:** ^1^ Key Laboratory of Cardiovascular Remodeling and Function Research, Chinese Ministry of Education and Chinese Ministry of Health, Department of of Cardiology, Qilu Hospital, Shandong University, Jinan, Shandong, 250012, China; ^2^ Laboratory of Experimental Teratology, Ministry of Education, School of Medicine, Shandong University, Jinan, Shandong, 250012, China; ^3^ Department of Genetics, School of Medicine, Shandong University, Jinan, Shandong, 250012, China

**Keywords:** atherosclerosis, inflammation, serum, proteomics, CDK9

## Abstract

Atherosclerotic coronary artery disease (CAD) is one of the most prevalent diseases worldwide. Atherosclerosis was considered to be the single most important contributor to CAD. In this study, a distinct serum protein expression pattern in CAD patients was demonstrated by proteomic analysis with two-dimensional gel electrophoresis coupled with mass spectrometry. In particular, CDK9 was found to be highly elevated in serum, monocytes and artery plaque samples of CAD patients. Furthermore, there was high infiltration of CD14+ monocytes/macrophages within artery plaques correlated with the expression of CDK9. Moreover, Flavopiridol (CDK9 inhibitor) could inhibit THP-1 cell (monocytic acute leukemia cell line) proliferation by targeting CDK9. Altogether, These findings indicate that CDK9 represent an important role for inflammation in the pathogenesis of atherosclerosis. It may be a potential biomarker of atherosclerotic inflammation and offer insights into the pathophysiology and targeted therapy for atherosclerotic CAD.

## INTRODUCTION

Coronary artery disease (CAD) is a leading cause of morbidity and mortality and has an increasing incidence worldwide [1]. Atherosclerosis was traditionally considered a metabolic disease characterized by the accumulation of lipids and fibrous elements in the large arteries, and constituted the single most important contributor to CAD [2, 3]. In the current view, accumulating evidences support a critical role for inflammation in the pathogenesis of atherosclerosis. Several studies have demonstrated that C-reactive protein [4, 5], B-type natriuretic peptide (NT-pro-BNP) [6], and serum cardiac troponin I (cTN-I) [7] as robust serum biomarkers for CAD risk.

Recent studies [8–10] have demonstrated that cyclin-dependent kinase 9 (CDK9) plays a crucial role in regulation of the cell cycle and monitoring the activation of primary inflammatory response genes. An increasing number of inhibitors, such as flavopiridol (FLA), have developed towards therapeutic applications in cancer and inflammation by targeting CDK9 [11–13]. Nonetheless, there were fewer researches for CDK9 in the atherosclerotic inflammation or atherosclerotic CAD.

In our previous study (unpublished data), results of the 2-D proteomics analysis revealed that CDK9 was highly expressed in the serum of patients with atherosclerotic CAD. Herein, the aim of this study was to analyze the expression levels of CDK9 in serum, monocytes, and plaque samples of atherosclerotic CAD patients and to explore the possible benefits in the prognosis and treatment of atherosclerotic CAD.

## RESULTS

### Proteomic analysis of serum samples of atherosclerotic CAD patients and healthy controls

**Figure 1:**
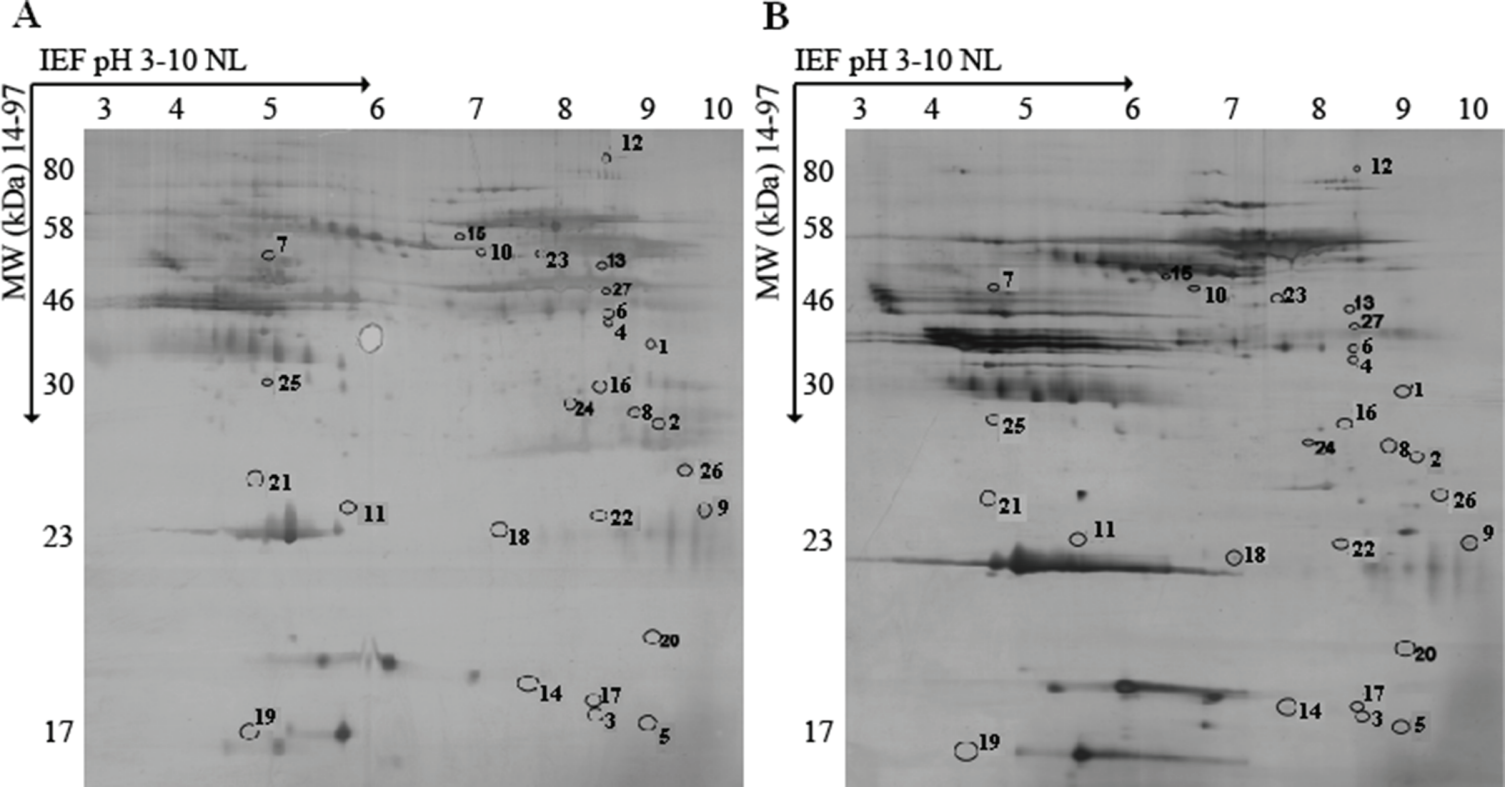
2-D electrophoretograms of serum sample (**A**) Atherosclerotic patients. (**B**) Healthy control subjects. The gels were silver stained and analyzed using PDQuest 2-D by Bio-Rad. Differentially expressed proteins are marked with *numbers* in the gel maps.

To identify aberrant serum proteins during atherosclerosis, we compared serum samples from atherosclerotic CAD patients and healthy control subjects. Figure 1 showed silver-stained 2-D electrophoresis IPG standard maps from one representative experiment with the two sample types. Spot analysis using 2-D PDQuest (Bio-Rad) detected 509 ± 31 spots per patient sample (Figure 1A) and 565 ± 29 spots per healthy control sample (Figure 1B). The protein expression was considered to have changed if the percentage volume of its spots on the gels showed a two-fold or greater difference (*P* < 0.05). Thirty-three differentially expressed proteins between the atherosclerotic CAD patient and healthy control were excised from the 2-D gels, digested in the gel and applied to a sample template for MALDI-TOF mass spectrometry. Twenty-seven protein spots were successfully identified with Mascot using peptide mass fingerprinting data. The protein names, NCBI accession numbers, theoretical molecular weight and pI values were shown in Table 1. Among 27 proteins identified, 15 of them, including CDK9, were increased, whereas 12 proteins were decreased in atherosclerotic serum samples.

**Table 1:** Identification of differentially expressed proteins in atherosclerotic patients compared with healthy controls

No.	Name	MW	pI	NCBI accession number
**Upregulated proteins**				
1	Angiotensin II receptor type-1 (clone HATR1GH) -human	41142	9.15	627378
2	T-cell receptor delta chain, CDR3 region	32819	8.96	4151024
4	Cyclin-dependent kinase 9	42777	8.97	4502747
7	Caspase 8 isoform E [Homo sapiens]	55391	4.99	15718712
8	Abhydrolase domain containing 10	33932	8.81	15778873
12	TNF receptor-associated protein 1	80109	8.3	23272132
13	Isocitrate dehydrogenase 2 (NADP+), mitochondrial precursor	50909	8.88	28178832
15	Phosphatidylinositol 4-phosphate 5-kinase type 2 alpha	61036	6.39	32167383
16	Ankynin repeat and SocS box-containing protein 7 isoform 2	36011	8.57	38176283
18	Interleukin 1 family, member 10	16943	4.94	74355171
23	NADH dehydrogenase (ubiquinone) Fe-S protein 4	52545	7.21	119573011
24	CD80 antigen (CD28 antigen ligand 1, B7–1 antigen)	33047	7.55	119599971
25	Cyclin D-type binding-protein 1	40261	4.71	119612996
26	Coenzyme Q4 homolog	29638	9.29	166795285
27	Mast cell carboxypeptidase A; MC-CPA	48669	9.08	317373331
**Downregulated proteins**				
3	ADP-ribosylation factor guanine nucleotide factor 6 isoform 6(ARF6)	19950	9.04	4502211
5	Chemokine-like factor isoform c	17169	9.41	7705933
6	WW45 protein	44633	9.12	10862824
9	G6B protein isoform G6b-C precursor	26147	9.68	19913377
10	Neuropilin-and tolloide-like protein isoform 1 precusor	57871	6.37	20452470
11	Glutathione S-transferase M5	23442	5.67	23065563
14	Interleukin-19 isoform 2 precursor	20451	7.62	30795208
17	Interleukin 20 precursor	20072	8.92	47481041
19	Interferon, alpha 14 [Homo sapiens]	22062	6.82	50960346
20	Mast cell-expressed membrane protein 1	21228	9.03	109698611
21	Dendritic cell-derived ubiquitin-like protein	26189	5.45	109896149
22	Tumor necrosis factor ligand superfamily member 8	26017	7.62	109896149

All 27 identified proteins were further classified into 6 different groups based on cell proliferation and apoptosis, inflammation factor, immune factor, energy metabolism and signaling pathway according to known or postulated functions and pathways (Table 2). CDK9 is the following emphasis of this study.

**Table 2:** Classification of known proteins

Category	Protein Name
Cell Cycle, Proliferation and Apoptosis	Cyclin-dependent kinase 9
	Caspase 8 isoform E [Homo sapiens]
	Phosphatidylinositol 4-phosphate 5-kinase type 2 alpha
	Cyclin D-type binding-protein 1
	WW45 protein
Inflammation Factor	Chemokine-like factor isoform c
	TNF receptor-associated protein 1
	Interleukin 1 family, member 10
	Interferon, alpha 14 [Homo sapiens]
Immune Factor	T-cell receptor delta chain, CDR3 region
	Interleukin-19 isoform 2 precursor
	Interleukin 20 precursor
	Mast cell-expressed membrane protein 1
	Dendritic cell-derived ubiquitin-like protein
	CD80 antigen (CD28 antigen ligand 1, B7–1 antigen)
	Mast cell carboxypeptidase A; MC-CPA
Energy Metabolism	Isocitrate dehydrogenase 2 (NADP+), mitochondrial precursor
	NADH dehydrogenase (ubiquinone) Fe-S protein 4
	ADP-ribosylation factor guanine nucleotide factor 6 isoform 6(ARF6)
	Neuropilin-and tolloide-like protein isoform 1 precusor
	coenzyme Q4 homolog
Signaling Pathway	Abhydrolase domain containing 10
	Glutathione S-transferase M5
	Ankynin repeat and SocS box-containing protein 7 isoform2
Other Categories	G6B protein isoform G6b-C precursor
	Tumor necrosis factor ligand superfamily member 8
	Angiotensin II receptor type-1 (clone HATR1GH) -human

### Validation of CDK9 expression in serum samples

**Figure 2:**
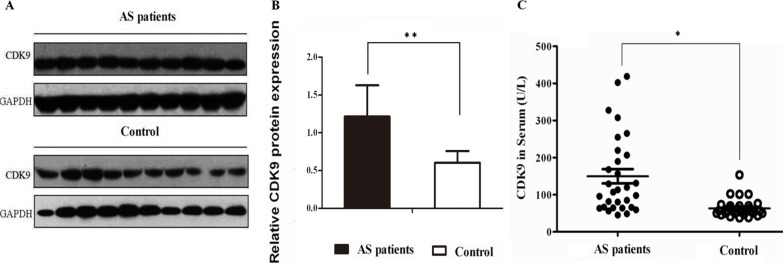
Validation of CDK9 expression in serum samples (**A**) Representative image of Western-blotting assay. (**B**) The relative expression of CDK9 protein (*Columns*, means; Bars, ± S.D.; **P < 0.01, 30 atherosclerotic patients, 25 healthy controls. (**C**) CDK9 level detected with ELISA (*Columns*, means; Bars, ± S.D.; *P < 0.05, 30 atherosclerotic patients, 25 healthy controls).

As shown in Figure 2A and 2B, CDK9 expression was found to be significantly increased in atherosclerotic patients compared with those in healthy controls (*P* < 0.01) in Western blotting assays. Figure 2C showed a 2.2-fold CDK9 increase in atherosclerotic serum samples measured with ELISA (*P* < 0.05, vs. Controls). Their characteristics (including proteomic analysis samples) are summarized in Supplementary Table S1.

### Validation of CDK9 expression in peripheral blood mononuclear cells (PBMCs)

**Figure 3:**
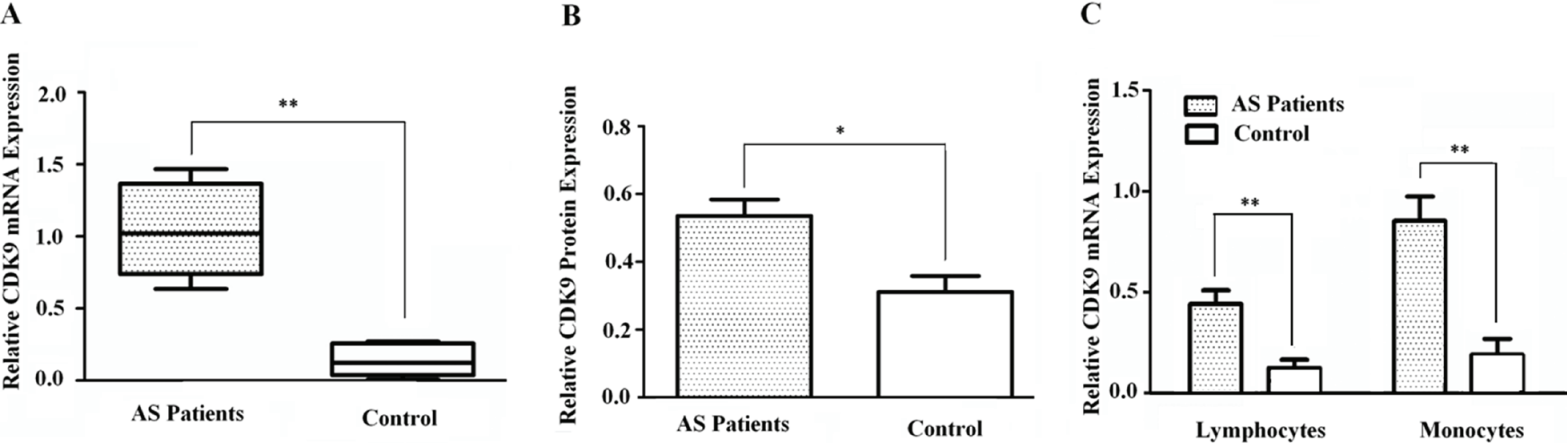
Validation of CDK9 expression in PBMCs (**A**) Elevated mRNA expression was found in atherosclerotic patients (*Columns*, means; Bars, ± S.D.; **P < 0.01; *n* = 5). (**B**) Consistent with mRNA expression, elevated CDK9 protein level was found in atherosclerotic patients (*Columns*, means; Bars, ± S.D.; *P < 0.05; *n* = 5). (**C**) Both lymphocytes and monocytes expressed CDK9, while monocytes showed higher levels than lymphocytes (*Columns*, means; Bars, ± S.D; **P < 0.01; *n* = 5).

We isolated PBMCs from atherosclerotic patients and healthy controls to measure CDK9 expression. As shown in Figure 3A and 3B, both mRNA and protein levels of CDK9 were found to be significantly increased in PBMCs of atherosclerotic patients compared with healthy controls. In addition, CDK9 was higher expressed in monocyte subpopulations than in lymphocyte subpopulations in PBMCs of atherosclerotic patients compared with healthy controls (*P* < 0.01, Figure 3C).

### CDK9 expression in atherosclerotic plaques

**Figure 4:**
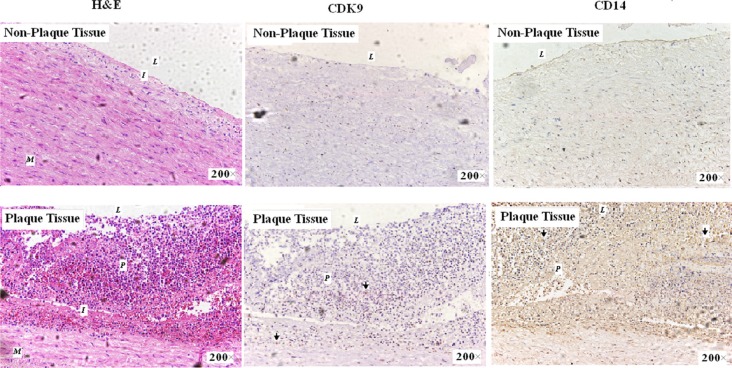
Immunohistochemistry staining of CDK9 and CD14 in artery wall sections (magnification 200×) H & E staining (left), CDK9 staining (middle) and CD14 staining (right). CDK9 expression was found mainly in nucleus on the area of plaque tissues (Arrow); CD14 expression was found mainly in cell membrane and cytoplasm on the area of plaque tissues (Arrow). M = Muscle; I = Intima; P = Plaque and L = Lumen.

In order to further investigate whether CDK9 was increased in atherosclerotic process, artery plaque tissue sections were analyzed by immunohistochemistry staining. As shown in Figure 4 (and Supplementary Figure 1), compared with non-plaque tissue, plaque tissue showed irregular intimal thickening, calcification, and significant atherosclerotic plaque formation, along with infiltration of abundant inflammatory cells. CDK9 positive expression was found in atherosclerotic plaque intima mainly located within nucleus. Furthermore, the CD14 (monocyte/macrophage surface marker) immunohistochemistry staining showed positive staining within atherosclerotic plaques which represented the majority of inflammatory infiltration cells. Moreover, the CD14^+^ cells showed increased CDK9 levels in atherosclerotic plaques, which indicated the role of CDK9 in monocyte infiltration during atherosclerosis.

### Inhibition of CDK9 expression by FLA in THP-1 cells

**Figure 5:**
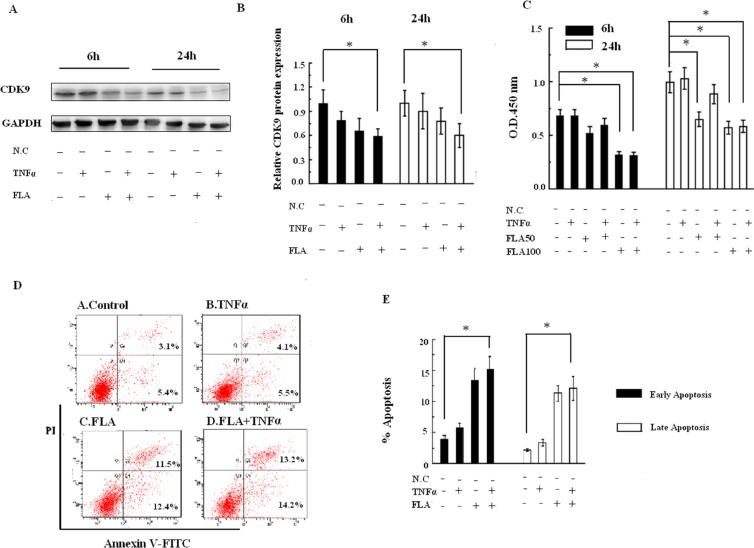
(**A**) Effects of FLA on the expression of CDK9 with or without TNFα stimuli. (**B**) The relative expression of CDK9 (*Columns*, means; Bars, ± S.D; *P < 0.05). (**C**) The CCK-8 assay showed that THP-1 cell proliferation was inhibited by FLA with or without TNFα stimulation. (**D**) Apoptosis measured by Annexin V/propidium iodide staining and flow cytometry. (**E**) Compiled percentage from three independent experiments of THP-1 cells treated with FLA with or without TNFα stimulation. *Columns*, means; Bars, ± S.D; *P < 0.05.

Because CDK9 was significantly increased in atherosclerotic patients and has been shown to be inhibited by FLA, the physiological properties of CDK9 treated with FLA were further investigated in THP-1 cells (human monocytic acute leukemia cell line). As shown in Figure 5A and 5B, CDK9 protein expression was decreased with FLA (100 nM) treatment combined with TNFα (50 ng/mL) stimulation for 6 h and 24 h.

To study the significance of the FLA-dependent suppression of CDK9 expression, THP-1 cells were treated with FLA (50 or 100 nM) combined with TNFα stimulation in CCK-8 assays. The dose of 100 nM FLA showed an apparent inhibitory effect at either 6 h or 24 h of TNFα-stimulated proliferation (Figure 5C). To determine the effect of CDK9 on monocyte apoptosis, flow cytometry analysis of THP-1 cells was performed with Annexin V-PI staining. As shown in Figure 5D and 5E, both the percentages of early and late apoptosis were significantly increased with FLA (100 nM) treatment combined with TNFα (50 ng/mL) stimulation. This result confirmed that FLA inhibits gene expression and potentiates tumor necrosis factor (TNF)-induced apoptosis.

In addition, we examined the cell cycle after FLA (100 nM) treatment with flow cytometry. As shown in Supplementary Figure 2, FLA treatment for 6 h might cause G2/M arrest (the percentage of G2/M cells from 10.9% to 12.6%). FLA treatment increased the percentage of G2/M cells to 15.2% under TNFα stimulation. However, there was no significant difference detected between groups for the percentage of cells in G_0_/G_1_ or S phase.

## DISCUSSION

The hallmark of the cyclin-dependent kinase is its ability to modulate cell cycle [14–16]. The member of the family, CDK9, was discovered by its ability involved in the control of gene transcription. The positive transcriptional elongation factor b (P-TEFb), which regulates the elongation phase of RNA-polymerase II-dependent transcription, is a heterodimer composed of CDK9 and cyclin T1 [17, 18]. Of note, dysfunctions in the CDK9-related pathway are related with several forms of human tumors [15, 19–24]. In addition, the roles of CDK9 in acquired immunodeficiency syndrome (AIDS) and cardiac hypertrophy have been reported [25–27].

Increasing evidences have shown that CDK9 is increased in activated lymphocytes, which has a critical role in inflammatory disorders [28–30]. In the present study, a distinct serum protein expression pattern in atherosclerotic CAD patients was demonstrated. In particular, CDK9 was found to be highly elevated in serum, monocytes and artery plaque samples of atherosclerotic CAD patients. Furthermore, there was high infiltration of CD14^+^ monocytes/macrophages within artery plaques correlated with the expression of CDK9. These findings indicated that CDK9 might represent an important role for inflammation in the pathogenesis of atherosclerosis. It may be a potential biomarker of atherosclerotic inflammation and provide incremental information regarding the prediction of CAD besides traditional risk factors. We merit further investigation of CDK9 measurement in various clinical situations for identifying coronary atherosclerosis.

In recent years, the anti-inflammatory effects of CDK9 inhibitors have been linked to its inhibition of cellular proliferation and induction of immune cell death [31]. Among these CDK9 inhibitors, FLA has shown a preference for inhibition of CDK9 over other CDKs [31]. FLA is an ATP analog that preferentially inhibits CDK9 kinase activity by a high-affinity interaction with its ATP-binding pocket [32]. FLA significantly inhibits inflammatory processes, such as dose-dependent murine collagen-induced arthritis [10, 33]; murine hepatitis induced by concanavalin A [34] and catabolic effects of pro-inflammatory cytokines on cartilage [35]. Our result showed that in THP-1 cells, FLA suppressed CDK9 expression, caused inhibition of proliferation and potentiation of TNFα induced apoptosis [36], demonstrated that FLA could inhibit THP-1 cell proliferation by targeting CDK9. But, it still needs many works to investigate possibility for the therapy of atherosclerotic CAD via CDK9 target.

## CONCLUSION

CDK9 was found to be highly elevated in serum, monocytes and artery plaque samples of atherosclerotic CAD patients. Also, there was high infiltration of CD14^+^ monocytes/macrophages within artery plaques correlated with the expression of CDK9. This indicated that CDK9 might be a potential biomarker of atherosclerotic inflammation and offer insights into the pathophysiology and targeted therapy for atherosclerotic CAD.

## MATERIALS AND METHODS

### Study population

Forty-three atherosclerotic CAD patients with symptoms of angina pectoris were included in the study after angiographic documentation of CAD from Qilu Hospital of Shandong University. Thirty-eight healthy volunteers without any evidence of atherosclerosis, CAD, hyperlipidemia, hypertension, diabetes mellitus or inflammatory disorders were enrolled from Jinan, Shandong Province, China and were used as the control group. This study was conducted in agreement with the Declaration of Helsinki and approved by the Human Ethics Committee of the School of Medicine, Shandong University.

### Serum collection, protein extracts and 2-D electrophoresis

An aliquot of 5 mL from each peripheral blood sample was separated into serum fractions by centrifugation at 1000 × *g* for 10 min. Serum protein was harvested with the Albumin and IgG Removal Kit (Pierce, Thermo Scientific, Rockford, IL, USA) according to the manufacturer's instructions. The supernatant was subsequently collected and used for 2-D electrophoresis. Approximately 300 μg of protein was suspended in a rehydration solution (8 M urea, 2% CHAPS, 65 mM DTT, 0.2% pharmalyte [pH range 3–10] and 0.2% bromphenol blue) and applied to 18 cm pH 3–10 non-linear Immobiline^™^ DryStrip Gels (GE Healthcare Bio-Sciences, Pittsburgh, PA, USA) for isoelectrofocusing [37]. The isoelectrofocusing was performed using an Ettan IPGphor^™^ Instrument (GE Healthcare Bio-Sciences), and proteins in the IPG strips were subsequently placed on a 12% uniform SDS-polyacrylamide gel. The gels were silver-stained and scanned with an Image Scanner in transmission mode, after which the image analysis was undertaken using 2-D PDQuest (Bio-Rad, Hercules, CA, USA).

### In-gel digestion and mass spetrometry analysis

The in-gel digestion of proteins for mass spectrometry analysis has been reported previously [37]. Peptide mass analysis was performed using an AB4800 MALDI TOF/TOF mass spectrometer (Applied Biosystems, Foster City, CA, USA) after dissolving the tryptic peptide mixture with 0.5% trifluoroacetic acid. The mass spectra were externally calibrated with a peptide standard from Applied Biosystems. Based on NCBI human databases, the mass spectra were analyzed with a 50 ppm mass tolerance by using GPS Explorer version 2.0.1 and Mascot version 1.9.

### Peripheral blood mononuclear cells (PBMCs) separation

Whole blood samples were collected from atherosclerotic CAD patients and healthy control groups. The blood was aspirated into sterile glass tubes with sodium citrate as an anticoagulant to facilitate PBMCs separation by Ficoll-Hypaque gradient centrifugation. Cells were washed twice with phosphate buffered saline to remove residual platelets. The remaining aliquot (200 μL) was placed into a microcentrifuge tube, frozen and kept at −80°C. The cold aggregation method was used with certain modifications to further separate the monocytes and lymphocytes [38]. Purity of the isolated monocytes was 90–95% as defined by flow cytometry with CD14^+^ staining.

### RNA isolation and RT-PCR

Total RNA was extracted from isolated PBMCs, lymphocytes and monocytes using the RNeasy Mini spin column kit (Qiagen, Inc., Valencia, CA, USA) according to the manufacturer's instructions. The RT-PCR was performed using the PrimeScript One Step RT-PCR kit version 2 (Takara Bio, Otsu, Japan). Sequences of specific primers for human CDK9 were 5′-GGAGACAGGGCATTTGAGTTA-3′ and 5′-ATAGGA TTGTGGGTGGGTGAG-3′, and those for GADPH as the internal control were 5′-AAGGTGAAGGTCGGAGTC AAC-3′ and 5′-GGGGTCATTGATGGCAACAATA-3′.

### Immunological assays procedures

The CDK9 level in serum sample was measured with Western blotting and ELISA (CDK9 antibody, SC-13130, Santa Cruz Biotechnology, Inc, Dallas, TX, USA; GAPDH antibody, HMM04, Sangong, Shanghai, China; Human CDK9 ELISA Kit E-12517, HuiJia Biotechnology, Xiamen, China).

Hematoxylin and eosin (H&E) staining of plaque tissue sections were performed according to standard procedures. Sections for immunohistochemistry staining were blocked sequentially with 3% hydrogen peroxide for 10 min and, 10% normal serum matching the host of the secondary antibody for 60 min at room temperature. Primary antibody against human CDK9 (1:800 dilution) or against human CD14 (1:100 dilution) was used. Later on, a standard rapid EnVision technique (REAL^™^ EnVision^™^ Detection System, Peroxidase/DAB+, Rabbit/Mouse, Code K5007, Dako, Denmark) was used to detect the protein conjugates and develop the color. The slides were viewed under 100 ×, 200 × and 400 × magnifications to define subcellular localization of the CDK9 and CD14.

### Proliferation and apoptosis analysis

The monocytic acute leukemia cell line THP-1 (American Type Culture Collection, Manassas, VA, USA) was maintained in RPMI 1640 media supplemented with 10% FBS at 37°C in a humidified atmosphere containing 5% CO_2_. A colorimetric cell proliferation assay using the CCK-8-kit (Dojindo Laboratories, Kumamoto, Japan) was carried out according to the manufacturer's instructions. Briefly, THP-1 cells at 1 × 10^5^ per well were cultured in 96-well plates and treated with different concentrations of FLA (50 and 100 nM) for 6 h and 24 h with or without TNFα (50 ng/mL) before measurement of O.D. 450 nm. To explore the effect of CDK9 on THP-1 apoptosis, FLA (100 nM) was separately added to the culture for 6 h with/without TNFα (50 ng/mL). Apoptosis was measured using the Annexin V-FITC apoptosis detection kit (BD Biosciences Pharmingen, San Diego, CA, USA) and analyzed with a FACSCalibur flow cytometer and CellQuest Pro software (Becton Dickinson).

### Statistical analysis

All continuous variables are expressed as means ± standard deviation (SD), unless stated otherwise. All tests were performed 2-sided and a significance level of *P* < 0.05 was considered to indicate statistical significance (*P < 0.05, **P < 0.01). All photo images of 2-D electrophoresis, Western blotting and immunohistochemistry staining represent at least three independent experiments.

## SUPPLEMENTARY FIGURES AND TABLES


