# Supplementary material for: Serum cyclin-dependent kinase 9 is a potential biomarker of atherosclerotic inflammation
Source: Oncotarget. 2015 Dec 1;7(2):1854–62. doi: 10.18632/oncotarget.6443 (PMC4811502; doi:10.18632/oncotarget.6443)
Supplement: Supplementary file 1 [file oncotarget-07-1854-s001.pdf]

Serum cyclin-dependent kinase 9 is a potential biomarker of atherosclerotic inflammation

Supplementary Materials

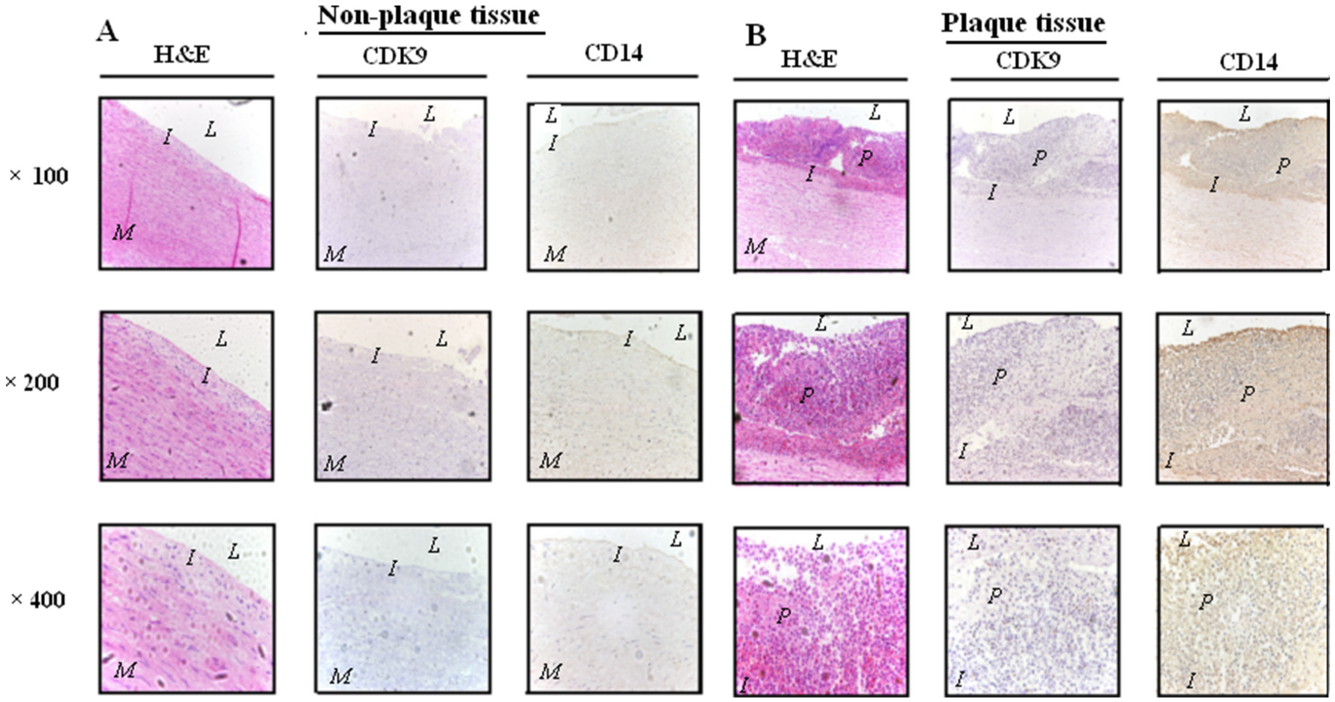

Supplementary Figure S1: Immunohistochemistry staining of CDK9 and CD14 in artery plaques tissue (magnification 100 × , 200 × and 400 ×). (**A**) Representative image of non-plaque tissue with H & E, CDK9 and CD14 staining. (**B**) Representative image of plaque tissue with H & E, CDK9 and CD14 staining. H & E staining (left), CDK9 staining (middle) and CD14 staining (right). M = Muscle; I = Intima; P = Plaque and L = Lumen.

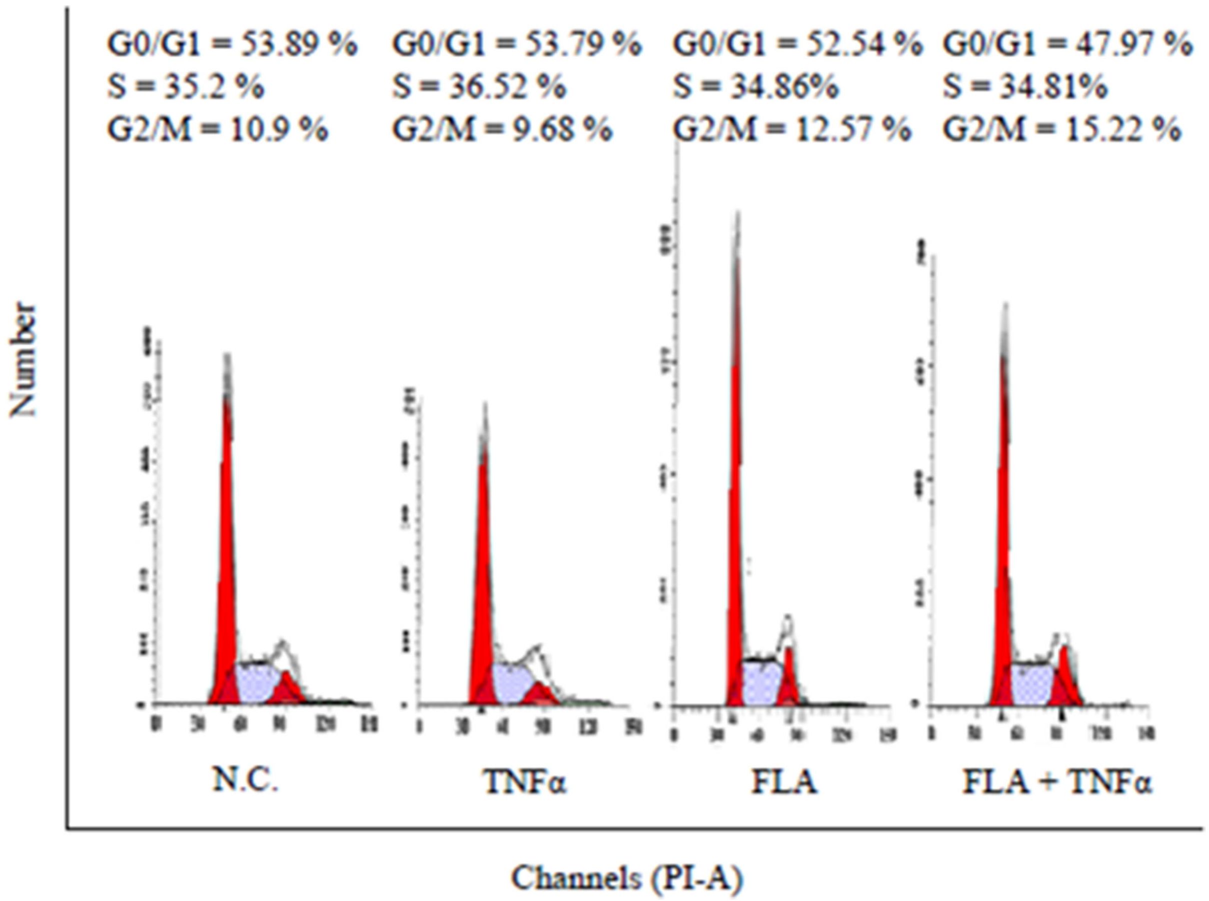

Supplementary Figure S2: Effects of FLA on the THP-1 cell cycle with or without TNFα stimulation.

Supplementary Table S1: Characteristics of CAD patients and healthy controls

**Characteristic**	**AS (*n* = 43)**	**Healthy control (*n* = 38)**
Age (years)	60 ± 3.3	58.0 ± 11.1
Gender(male/female) (n)	25 /18	17/21
Current smoker (n)	22 (51.16%)	17 (44.74%)
Hypertension (n)	30 (69.76%)	5 (13.15%)
Systolic pressure (mmHg)	147.64 ± 26.17 *	125.88 ± 9.77
Diastolic pressure (mmHg)	91.70 ± 15.93*	75.47 ± 6.48
Hyperlipidaemia (n)	31 (72.09%)	8 (21.05%)
TC (mmol·L -1)	5.21 ± 1. 51*	4.59 ± 0.76
TG (mmol·L -1)	1.40 ± 1.92*	1.13 ± 0.23
HDL -C (mmol·L-1)	1.29 ± 0.41*	1.52 ± 0.32
LDL-C (mmol·L-1)	3.21 ± 0. 96*	2.93 ± 0.69
CAD (n)	43	0
One-vessel disease	28 (65.11%)	
Two-vessel disease	12 (27.91%)	
Three-vessel disease	3 (6.97%)	
Blood Glucose (/mmol·L-1)	6.35 ± 2.07*	4.92 ± 0.56

Results are presented as mean ± S.D., median (range) or number (%) of patients. TC: Total TC: Total Cholesterol; TG: Total Triglyceride; HDL-C: high density lipoproteins cholesterol; LDL-C: low density lipoprotein cholesterol. *P < 0. 05.
